# Interaction of Carrier Protein with Potential Metallic Drug Candidate *N*-Glycoside ‘GATPT’: Validation by Multi-Spectroscopic and Molecular Docking Approaches

**DOI:** 10.3390/molecules26216641

**Published:** 2021-11-02

**Authors:** Sabiha Parveen, Mohd. Sajid Ali, Hamad A. Al-Lohedan, Sartaj Tabassum

**Affiliations:** 1 Department of Chemistry, Aligarh Muslim University, Aligarh 202002, India; sabihap1@gmail.com; 2 Department of Chemistry, College of Sciences, King Saud University, Riyadh 11451, Saudi Arabia; smsajidali@gmail.com (M.S.A.); hlohedan@ksu.edu.sa (H.A.A.-L.)

**Keywords:** lysozyme, organotin, interaction studies, thermodynamics, molecular docking

## Abstract

Lysozyme is often used as a model protein to study interaction with drug molecules and to understand biological processes which help in illuminating the therapeutic effectiveness of the drug. In the present work, in vitro interaction studies of 1-{(2-hydroxyethyl)amino}-2-amino-1,2-dideoxy-d-glucose triphenyl tin (IV) (GATPT) complex with lysozyme were carried out by employing various biophysical methods such as absorption, fluorescence, and circular dichroism (CD) spectroscopies. The experimental results revealed efficient binding affinity of GATPT with lysozyme with intrinsic binding (K_b_) and binding constant (K) values in the order of 10^5^ M^−1^. The number of binding sites and thermodynamic parameters ΔG, ΔH, and ΔS at four different temperatures were also calculated and the interaction of GATPT with lysozyme was found to be enthalpy and entropy driven. The CD spectra revealed alterations in the population of α–helical content within the secondary structure of lysozyme in presence of GATPT complex. The morphological analysis of the complex with lysozyme and lysozyme-DNA condensates was carried out by employing confocal and SEM studies. Furthermore, the molecular docking studies confirmed the interaction of GATPT within the larger hydrophobic pocket of the lysozyme via several non-covalent interactions.

## 1. Introduction

The interaction studies of small molecules with proteins are of great importance in determining the ADME properties of a drug molecule with direct implications on its pharmacokinetics and pharmacodynamics [1,2,3,4,5,6,7]. The interaction of the drug with proteins can strongly affect the rate of drug distribution as well as elimination. Therefore, these interaction studies are promising for the interpretation of the mechanisms involved in the metabolism and transportation of the drug to the target site [8].

Lysozyme is a globular enzymatic protein that can be found in various secretions such as human tears, saliva, mucus, milk, serum, and cerebrospinal fluid [9]. Lysozyme is abundant in egg white protein; ~3.5% protein content is occupied by lysozyme [10]. Hen egg-white lysozyme (HEWL) can be used as a model protein for studying drug-protein interactions due to its similarity with human lysozyme [11,12]. HEWL is composed of two major domains: (i) α-domain, consisting of α-helices, and (ii) β-domain, consisting of an anti-parallel β-sheet. Lysozyme exhibits diverse pharmacological properties like anti-bacterial, anti-cancer, anti-viral, anti-septic, and anti-inflammatory. It is also known to bind reversibly with small molecules and has been reported to be used as a carrier in drug delivery [13,14,15,16,17].

The combinatorial approach to coordinate metal ion with the organic scaffold offers several advantages over the conventional organic compounds in the drug discovery such as better reactivity, lipophilicity, reduce drug dosage, better cellular uptake, and reduced systemic toxicity [18,19]. In this lieu, the rapidly advancing field of glycobiology has stimulated great interest in inorganic medicinal chemistry. Carbohydrates are of much interest for drug designing because (i) of their high water solubility, (ii) better cellular uptake due to lipophilicity which also helps in facile transport at the molecular level, (iii) they can act as active sites for molecular recognition, and (iv) they are most abundantly available and represent the key species in several biological processes of living organisms. Therefore, these bioactive molecules on coordinating with metal ions could enhance their activity and compatibility and serve as promising candidates for drug designing [20,21].

Metal complexes have gained much attention in drug discovery, being widely employed as therapeutic compounds to treat various human diseases such as diabetes, neurological disorders, cancer, etc. Metals exhibit unique characteristics that include variable coordination modes, redox activity, reactivity towards organic substrates for which metal complexes, either drugs or pro-drugs, have become potential candidates in medicinal chemistry [22,23,24,25,26,27,28,29]. Numerous researches have shown remarkable progress in the utilization of organometallic complexes as drugs to treat various diseases as they exhibit modified pharmacological and toxicological properties [30]. Since these complexes exhibit kinetic stability, relative lipophilic character, structural diversity, great ability to bind to biological targets, they provide ample opportunities in drug designing as compared to ‘coordination complexes’ [31,32]. The use of organotin complexes as therapeutic compounds has become more and more pronounced in drug designing because of their remarkable pharmacological profile. Organotin complexes exhibit attractive properties such as (i) they can bind to glycoproteins and can also interact with DNA directly, leading to cell death by apoptotic mechanisms, (ii) increased water solubility, and (iii) better body clearance. The pharmacological activity of organotin complexes is significantly affected by the hydrolyzable groups linked to the tin atom that controls the delivery of the active organotin ions. The organic moiety attached to the tin atom directs the transport across the cell membrane and the coordination position at the tin atom [33,34,35,36,37].

In this work we are reporting interaction studies of lysozyme with 1-{(2-hydroxyethyl)amino}-2-amino-1,2-dideoxy-d-glucose triphenyl tin (IV) (GATPT) complex which was previously synthesized in our laboratory [38] by adopting a combinatorial approach involving a combination of *N*-glycoside (carbohydrate scaffold) with triphenyltin (IV) moiety, which modulates the pharmacological parameters of both the fragments pertinent to the delivery of a drug to the cells. The detailed investigation of interaction studies of GATPT with lysozyme was carried out by employing various complementary techniques. The binding affinity was ascertained by UV-vis and fluorescence studies. Conformational changes in the secondary structure were determined by CD spectroscopy. Morphology of the complex with lysozyme and lysozyme-DNA condensates was studied by scanning electron and confocal microscopy. Furthermore, molecular docking studies were performed to validate the mode of interactions.

## 2. Results and Discussions

### 2.1. Conformational Changes in Lysozyme Investigated by UV–Visible Spectroscopy

**Figure 1:**
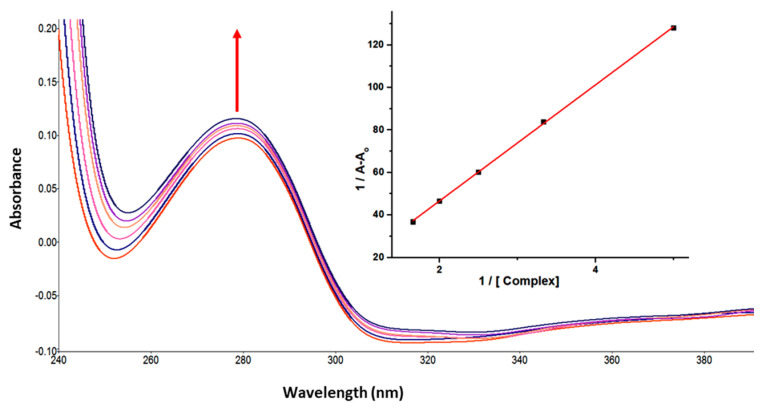
Absorption spectra of lysozyme in tris-HCl buffer on progressive addition of GATPT at 25 °C. Inset: plot of 1/A−A_o_ vs. (complex). [Lyso] = 10 µM, (GATPT) = 0–50 µM.

UV-vis absorption spectroscopy is one of the basic methods for studying the structural changes in biomacromolecules and investigating ligand-protein interactions. The UV-vis spectrum of GATPT exhibited strong absorption around ~270 and 380 nm corresponding to π−π* and n−π* transitions, respectively. Lysozyme, on the other hand, shows an absorption band at ~280 nm owed to π–π* transitions due to the presence of three amino acids, viz, tryptophan, tyrosine, and phenylalanine [39]. On progressive titration of GATPT (0–50 µM) to constant concentration of lysozyme (10 µM) (Figure 1), ‘hyperchromism’ was observed at ~280 nm corresponding to non-covalent interactions. Hyperchromism is usually associated with the breakage of the hydrogen bonds leading to the conformational alteration in the secondary structure of the lysozyme. The quantitative assessment of binding affinity of GATPT towards lysozyme was done by calculating intrinsic binding constant, *K*_b_ by employing equations given in ESI and was found to be 2.9 × 10^5^ M^−1^. The changes in the absorption spectra of lysozyme upon addition of GATPT provide an inference that the microenvironments around three amino acid residues including the secondary structure of lysozyme were altered due to the binding of GATPT.

### 2.2. Intrinsic Fluorescence Measurements

**Figure 2:**
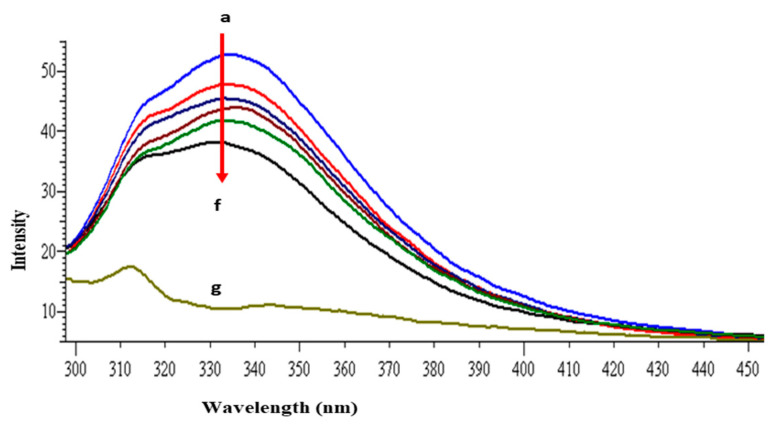
Emission spectra of lysozyme in tris–HCl buffer in the presence of varying amounts of GATPT complex at 298 K (GATPT) 0–50 µM. (a) represents lysozyme alone, (b–e) are intensity changes upon increasing concentration of GATPT with lysozyme, (f) represents DNA-lysozyme with GATPT, and (g) is GATPT alone.

The intrinsic fluorescence property of lysozyme is mainly because of the presence of tryptophan and tyrosine amino acid residues [40]. When the protein is excited at 280 nm the resultant fluorescence emission around 340 nm is due to the tryptophan and tyrosine residues, whereas at 295 nm excitation wavelength, fluorescence intensity is solely due to the tryptophan residue. The other fluorophore present in the lysozyme is phenylalanine but with very weak and negligible fluorescence [41]. Lysozyme consists of six tryptophan residues; four reside in the α-domain whereas two residues are in the loop region, connecting the α- and β-domains. Out of the total of six tryptophan residues, Trp62 and Trp108 are slightly exposed to the solvent and are primarily responsible for exhibiting intrinsic fluorescence [42]. Figure 2 shows the intrinsic fluorescence spectra of lysozyme with and without GATPT at 295 nm excitation wavelength. The successive reduction in fluorescence intensity and wavelength maxima were observed on the progressive addition of GATPT to lysozyme which could be attributed to the binding of GATPT in the vicinity of the Trp fluorophores (Trp62 and Trp108) of lysozyme [43,44]. An interesting observation was the reduction of emission intensity on the addition of DNA-lysozyme to GATPT which was even less than lysozyme alone and lysozyme +GATPT complex. The above observations could reveal the involvement of certain biochemical interactions between GATPT and lysozyme and then GATPT leaving lysozyme and interacting with DNA. To minimize the inner filter effect, a low complex concentration has been used and further corrected as per the methods reported in literature [40]. The binding constant *K* was determined by using modified Stern-Volmer equation (Equation (S7)) [45] and was found to be 2.32 × 10^5^ M^−1^. Further, binding constants were also determined at three other different temperatures (303, 308, 313 K) and values are shown in Table 1.

Moreover, the dissociation constant (K_D_) values were also calculated which is a useful way to present the affinity of a drug molecule for its biological target. GATPT exhibited impressive dissociation constant values at all four different temperatures (given in Table 1). It is interesting to mention that the complex exhibited better dissociation constant values as compared to two well known drugs, viz, cisplatin and oxaliplatin, which exhibit K_D_ values as 1.83 ± 0.03 and 1.51 ± 0.02 mM, respectively.

### 2.3. Thermodynamic Parameters and Binding Modes

**Figure 3:**
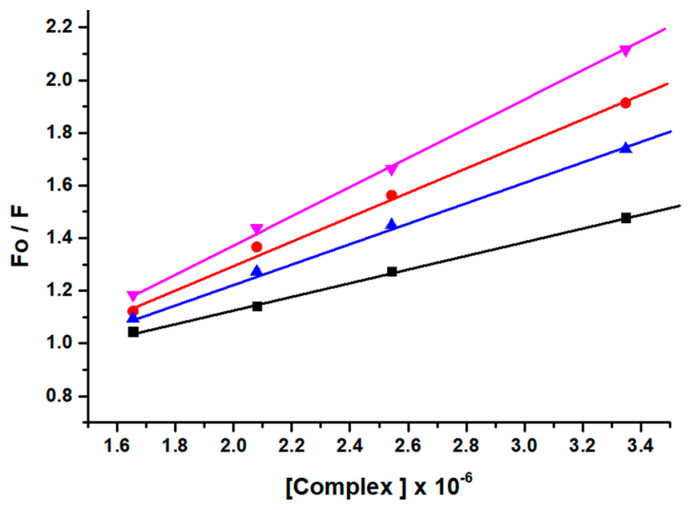
Stern−Volmer plots corresponding to fluorescence quenching of lysozyme in presence of complex GATPT at four different temperatures, 298 (black), 303 (red), 308 (blue) and 313 K (pink).

The mechanism of fluorescence quenching was ascertained by performing fluorescence quenching experiments at four different temperatures (298, 303, 308, and 313 K) and the fluorescence quenching data were examined by using the linear Stern–Volmer Equation (S6) [46] as given in Figure 3.

The results obtained demonstrated that the Stern–Volmer quenching constant K_SV_ was in inverse relation to the temperature and k_q_ (10^13^ M^−1^ s^−1^) is higher than the limiting diffusion constant K_dif_ of the biomolecule (K_dif_ = 2.0 × 10^10^ M^−1^ s^−1^) which is consistent with the static quenching mechanism [47].

Furthermore, the fluorescence quenching experiment was effectively utilized to determine the binding sites (*n*) and binding constant (*K*) (Table 1) from the double logarithm Equation (S7).

The number of binding sites (*n*) were calculated to approximately equal to 1 at all four temperatures, which strongly supported the existence of a single binding site for the complex GATPT with lysozyme.

The values of thermodynamic parameters, viz enthalpy change (∆H), entropy change (∆S), and free energy change (∆G), are the main evidence for ascertaining the binding modes and were calculated from Van’t Hoff Equations (S8) and (S9) [48,49,50,51].

**Figure 4:**
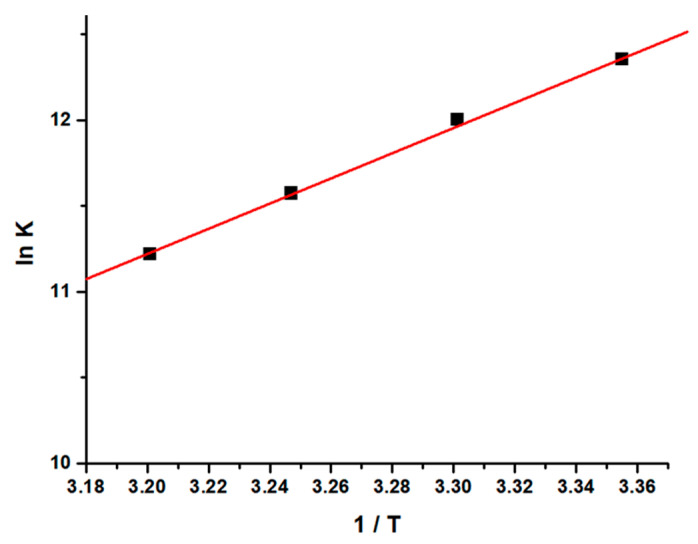
Van’t Hoff plot for the interaction of lysozyme and complex GATPT.

From the thermodynamic standpoint, ∆H > 0 and ∆S > 0 corresponds to hydrophobic interactions; ∆H < 0 and ∆S < 0 implies the van der Waals forces or hydrogen bond formation, whereas ∆H ≈ 0 and ∆S > 0 suggests the electrostatic forces. The positive ∆S value is usually taken as an evidence for hydrophobic interactions [52,53,54]. Figure 4 shows the variation of ln [*K*] as a function of 1/T. As evident from the Table 1, the negative ∆H and positive ∆S values, as estimated from the slope and intercept of the linear regression plot, are suggestive of hydrophobic and hydrogen bond interactions which play major roles in the GATPT-lysozyme binding reaction and contribute to the stability of the complex.

### 2.4. Conformational Transitions Monitored by CD

**Figure 5:**
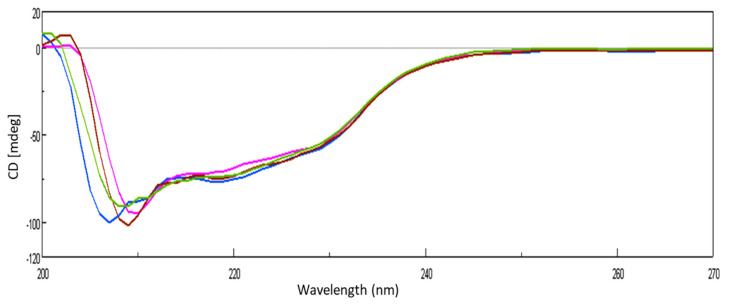
CD spectra of lysozyme in the absence and presence of different concentrations of GATPT at 25 °C, (lysozyme) = 10^−5^ M (blue color), GATPT (brown, green, pink color: 5, 10, 20 µM respectively).

The conformational transition of lysozyme on interaction with GATPT was studied by employing circular dichroism (CD) spectroscopy. CD titrations were carried out as a function of GATPT concentration to analyze the conformational changes in lysozyme and binding sites of the lysozyme upon titration with GATPT. The observed CD spectra of lysozyme obtained before incubation exhibited the typical spectrum of predominantly α-helical conformation and in presence of GATPT (5, 10, 20 µM), there was a decrement in the ellipticity of lysozyme (Figure 5). An intense signature negative peak was observed at 208 nm and another band was observed at 222 nm corresponding to π−π* and n−π* transitions for both α-helix and random coil, respectively. On incubation of lysozyme-GATPT, there was an anticipated reduction in the negative value at 208 nm, which was suggestive of the interaction of GATPT complex with the amino acid residues of the polypeptide chain and disruption of the hydrogen bonding in the lysozyme leading to the alteration of the secondary structure of the lysozyme [55]. The α-helix content of free lysozyme was observed to be 26.33% which decreased in presence of GATPT (5–20 µM) to 24.66–25.78%. This decrease in the α-helix content of lysozyme in presence of GATPT suggests that GATPT interacted with lysozyme mostly in the α-helix region that results in the alteration of the secondary structure of the protein [56,57]. Deconvulated spectral analysis was also carried out by converting CD (mdeg) data into MRE values; the MRE spectra are provided in ESI Figure S1.

### 2.5. Binding Location of GATPT within the Lysozyme: Molecular Docking Studies

**Figure 6:**
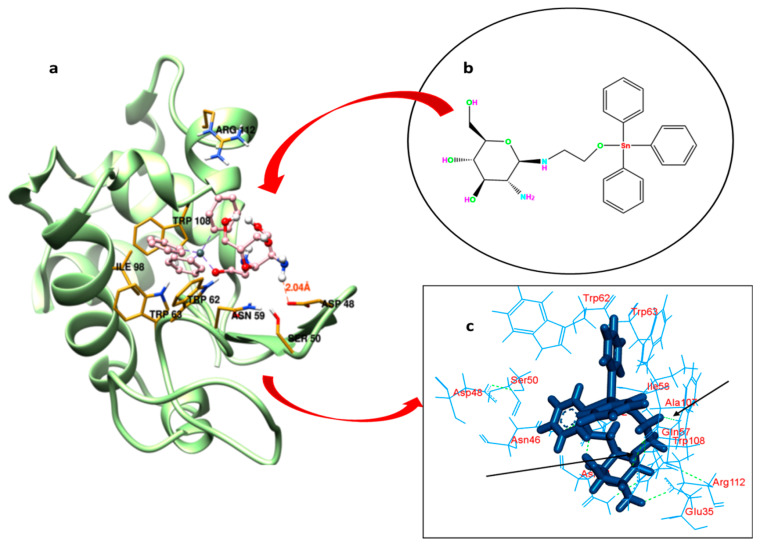
Best fit molecular docked pose of GATPT at the active site of lysozyme (**a**), structure of GATPT complex (**b**) and amino acid residues of lysozyme involved in the interaction with GATPT and arrows showing hydrogen bonding interactions between GATPT and amino acid residues (**c**).

Understanding the binding locus of a small molecule or drug inside the carrier protein is of imperative importance to predict the efficacy of the latter in the biology and medicine [58]. GATPT (structure shown in Figure 6b) binding sites within the lysozyme have been explored by carrying out molecular docking studies. The possible pockets of lysozyme are presented in Figure 6a. The big hydrophobic cavity of lysozyme played an important role in the binding because this is the gateway to reach the GATPT complex to the active site; further, it was revealed from molecular docking that the proposed binding site of GATPT within the lysozyme was located in the cleft region near the catalytic site of the macromolecule [59]. The hydrophobic pocket comprises of several amino acid residues which are important for the binding perspective and among these Trp62 is fully exposed residue while Trp 63 is less exposed in comparison to the former. Other residues such as Trp108 and Ala107 are also important residues present in that pocket. The non-covalent interactions that exist in the binding of GATPT and lysozyme were hydrophobic forces and hydrogen bonding (Figure 6c). Most of the residues interacted through the hydrophobic forces and these were Arg112, Gln57, Ala107, Ile58, Asn44, Asn46, Asp48, Asp52, Trp62, Trp63, Trp108, and Ser50 (Table 2), and the residues bound through the hydrogen bonding were Glu35 and Asn46. It is important to note here that all three Trp residues (62, 63, and 108) play an important role in the complexation of GATPT with the lysozyme. Table 2 represents the hydrophobic interactions between amino acid residues of the lysozyme and GATPT.

### 2.6. Confocal Laser Scanning Microscopy of the Complex and Lysozyme and Lysozyme-DNA Condensate

**Figure 7:**
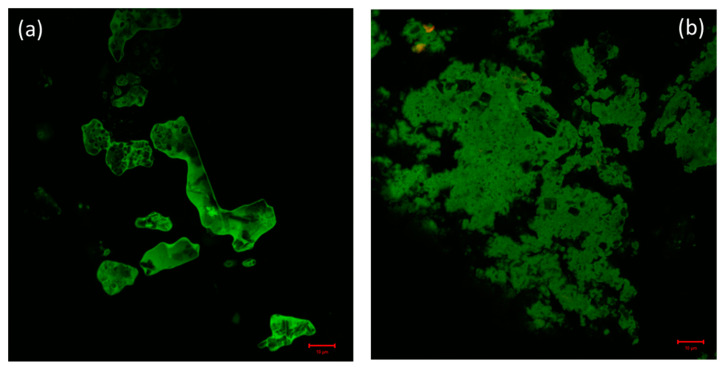
(**a**) Confocal image of GATPT-Lysozyme complex and (**b**) confocal image of GATPT- Lysozyme-DNA complex.

Confocal microscopy is a very powerful technique used for the determination of the distribution of protein molecules and morphological changes in condensates of drug and biomolecules [60]. By exploiting confocal microscopy we analyzed the formation and the distribution of GATPT-lysozyme and GATPT-lysozyme-DNA complexes. Figure 7a shows GATPT-lysozyme condensate and Figure 7b shows GATPT-lysozyme-DNA condensate. The observed results exhibited morphological transitions in GATPT-lysozyme condensate as it was incubated with DNA, suggestive of GATPT uptake by DNA. It could be inferred that some part of the drug (GATPT) molecule is getting transferred from lysozyme to DNA and lysozyme is acting as a carrier to deliver GATPT to the DNA target. The confocal results were consistent with the results of SEM.

### 2.7. Scanning Electron Microscopy (SEM)

**Figure 8:**
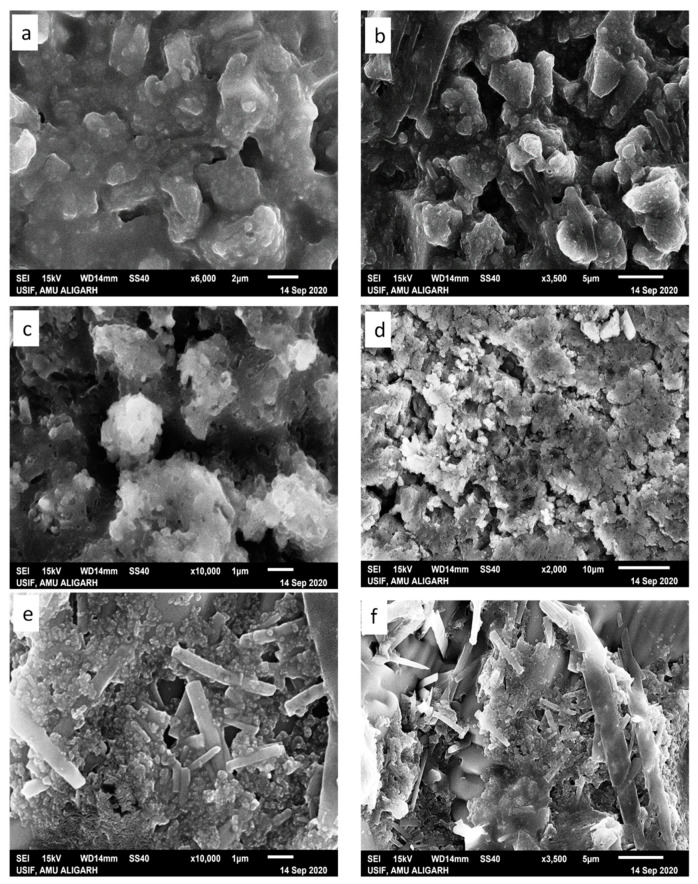
SEM images of GATPT complex (**a**,**b**), condensate of GATPT-lysozyme (**c**,**d**), and condensate of GATPT-lysozyme-DNA (**e**,**f**) incubated after 24 h.

Literature reports reveal that the morphology of the condensate primarily depends on the type of ion, ionic strength, solvent polarity, and nature of the condensing agent (charge density and surface of the substrate) [61]. Herein, condensates (i) lysozyme-GATPT and (ii) lysozyme-DNA-GATPT were prepared by evaporating equimolar mixtures of lysozyme, DNA, and GATPT, under neutral conditions in aqueous Tris–HCl buffer (50 mM NaCl, pH 7.2). SEM and SEM-EDX was employed to view and analyze morphological changes of the condensates (i) and (ii) in comparison to the GATPT alone. Micrographs of GATPT shown in Figure 8a,b displayed irregular crystalline morphologies of variable sizes and shapes. However, the SEM micrographs of GATPT-lysozyme displayed the amorphous clusters where the lysozyme molecules were found to be converted into dissocial, mesh-like structures (Figure 8c,d), considered to be loosely bound by colloidal particles and hydrophobic interactions in an unordered fashion. Amorphous clusters usually undergo energy orientation and some conformational alterations because of changes in multiple H-bonds and disulfide bonds that play a critical role in stabilizing the structure [62], leading to the loss of the alpha helical content of the lysozyme. The micrographs of condensate (ii), i.e., GATPT-lysozyme-DNA, depicted morphological transition evidenced by the formation of rectangular and concrete-like structures indicating the condensation of DNA and lysozymes into compact, massive structures (Figure 8e,f). The observed results displayed more prominent morphological changes with condensate (ii) having DNA as compared to condensate (i) suggesting the affinity of GATPT for DNA leaving lysozyme; therefore, it can be concluded that lysozyme can act as a good carrier molecule to deliver the drug to DNA target.

## 3. Conclusions

Pertinent to the important pharmacological characteristics of lysozyme in various therapies and its interaction with small molecules, in vitro binding studies with GATPT complex were carried out by employing multi-spectroscopic techniques such as UV-vis, fluorescence, and CD. To quantify the binding strength of GATPT with lysozyme, intrinsic binding (*K*_b_) and binding constant (*K*) values were calculated which revealed impressive binding propensity in the order of 10^5^ M^−1^. Interaction occurred via non-covalent, hydrophobic interactions and hydrogen bonding. Secondary structure of lysozyme was also altered upon interaction with GATPT which was evidenced from the decrease in α–helical content of lysozyme. The confocal analysis revealed that these studies help to identify specific drug-protein interactions and the conformational alterations in protein structure upon interaction with GATPT.

## Figures and Tables

**Table 1:** Binding and thermodynamic parameters of lysozyme binding with GATPT at four different temperatures (298, 303, 308, 313 K).

T (K)	*K* (10^5^ M^−1^)	*K*_sv_ (10^5^ M^−1^)	K_D_ (µM)	Binding Site (*n*)	∆H (kJ mol^−1^)	∆S (J k^−1^ mol^−1^)	∆G (kJ mol^−1^)
298	2.32	4.05	4.31	0.932	−59.46	92.06	−86.89
303	1.63	3.32	6.13	1.010			−87.35
308	1.06	1.39	9.43	1.014			−87.81
313	0.76	0.89	13.15	1.056			−88.274

**Table 2:** Molecular docking parameters of lysozyme-GATPT interaction.

Amino Acid Residues	Interaction Involved
Glu35	Hydrogen bonding
Asn46	
Arg112	Hydrophobic
Gln57	
Ala107	
Ile58	
Asn44	
Asn46	
Asp48	
Asp52	
Trp62	
Trp63	
Trp108	
Ser50	

## Data Availability

Study does not include data.

## Supplementary Materials

The following are available online. Figure S1: Mean residual ellipticity (MRE) spectra of lysozyme in the absence and presence of different concentrations of GATPT at 25 °C, (Lysozyme) = 10^−5^ M (orange color), GATPT (grey, blue, red color: 5, 10, 20 µM respectively).
