# Supplementary material for: Interaction of Carrier Protein with Potential Metallic Drug Candidate N-Glycoside ‘GATPT’: Validation by Multi-Spectroscopic and Molecular Docking Approaches
Source: Molecules. 2021 Nov 2;26(21):6641. doi: 10.3390/molecules26216641 (PMC8587009; doi:10.3390/molecules26216641)
Supplement: Supplementary file 1 [file molecules-26-06641-s001.zip › molecules-1415435-supplementary.pdf]

Electronic Supplementary Information

Interaction of Carrier Protein with Potential Metallic Drug candidate N-Glycoside 'GATPT': Validation by Multi-spectroscopic and Molecular Docking Approaches

Sabiha Parveen[a], Mohd. Sajid Ali[b], Hamad A. Al-Lohedan[b], Sartaj Tabassum[a]*

[a]Department of Chemistry, Aligarh Muslim University, Aligarh 202002, India.

[b]Department of Chemistry, College of Sciences, King Saud University, Riyadh 11451, KSA.

Corresponding Author. Tel.: +91 5712703893.

E–mail address: tsartaj62@yahoo.com

Experimental section

Reagents and materials

GATPT complex was previously synthesized and thoroughly characterized in our laboratory [1]. Hen egg white lysozyme (HEWL) was purchased from Sigma Aldrich. A stock solution of lysozyme was made in 20mM Tris HCl buffer, pH 7.4 and its concentration was estimated by using UV-visible spectrophotometer (Perkin Elmer Lambda 25) using molar extinction coefficient of 38940 cm^{-1} at 280 nm [2].

Absorption spectra were recorded on a Perkin–Elmer Lambda 25 using cuvettes of 1 cm path length, and the data were reported in λ_{max}/nm.

The confocal images were taken by laser scanning confocal microscope, Zeiss LSM 780 with airyscan detector. The samples were excited at 488 nm.

Emission intensity measurements were carried out using Hitachi F–2700 fluorescence spectrophotometer in a 1 cm path length quartz cell. The duration taken to obtain fluorescence emission spectra was approximately 3 minutes per set which took overall 30 minutes for 10 sets as reported in emission spectra.

In vitro interaction studies with lysozyme

Experiments involving interaction of GATPT complex with lysozyme were carried out in 5 mM Tris–HCl, buffer, pH = 7.4. The binding experiments including absorption spectral traces with lysozyme conformed to the standard methods [3].

By assuming that there is only one type of interaction between the complex GATPT and lysozyme in aqueous solution, eqn (1) and (2) can be established:

$$[Lyso] + [Complex] \leftrightarrow Lyso{:}Complex \qquad (1)$$

$$K_b = \frac{[Lyso:Complex]}{[Lyso][Complex]} \qquad (2)$$

where K_b is the binding constant for the complex GATPT

Assuming [Lyso: Complex]= C_B

$$K_b = \frac{C_B}{(C_{Lyso} - C_B)(C_{Complex} - C_B)} \qquad (3)$$

where C_{Lyso} and $C_{complex}$ are analytical concentration of lysozyme and complex in the solution, respectively. According to the Beer–Lambert law

$$C_{Lyso} = \frac{A_O}{\varepsilon_{Lyso} l} \qquad (4)$$

$$C_B = \frac{A - A_O}{\varepsilon_{Lyso} l} \qquad (5)$$

Where A_o and A are the absorbance of lysozyme at 280 nm, in the absence and presence of complex, respectively. ε_{Lyso} and ε_B are the molar extinction coefficient of lysozyme and the bound complex, respectively, and **1** is the light path of the cuvette (1 cm).

By substituting ε_{Lyso} and ε_B in eqn (4) and (5) in eqn (3), the equation can be deduced as follows:

$$\frac{A_o}{A - A_o} = \frac{\varepsilon_{Lyso}}{\varepsilon_B} + \frac{\varepsilon_{Lyso}}{\varepsilon_B K} \frac{1}{C_{Complex}}$$

Thus, the double reciprocal plot of 1/ A–Ao *vs.* $1/C_{complex}$ is linear and the binding constant can be estimated from the ratio of the intercept to the slope.

Thermodynamic parameters and binding modes

The fluorescence quenching data were examined at four different temperatures (298, 303, 308, and 313 K) by using the linear Stern–Volmer equation (6)

$$F_0/F = K_{sv}+1 = k_q\tau_o [Q] +1 \qquad (6)$$

where Fo and F are fluorescence intensities in the free and bound form, respectively, where K_q corresponds to the biomolecular quenching rate constant and τ_o represents the average lifetime of the fluorophores in absence of quencher and its value is around 10^{-8} s for most of the biomolecules, [Q] is the concentration of the quencher.

The binding sites (n) and binding constant (K) were determined from the double logarithm equation 7

$$\text{Log } (F_0/F - 1) = \log K + n \log [Q] \qquad (7)$$

After plotting $\log(F_0 - F/\,F)$ against $\log[Q]$, the slope obtained gave the number of binding sites (n).

The values of thermodynamic parameters viz., enthalpy change (ΔH), entropy change (ΔS) and free energy change (ΔG) are the main evidence for ascertaining the binding modes and were calculated from Van`t Hoff equations (8& 9) as

$$\ln K = \frac{-\Delta H}{RT} + \frac{\Delta S}{R} \qquad (8)$$

$$\Delta G = \Delta H - T\Delta S = -RT \ln K \qquad (9)$$

where R is the universal gas constant, T is the temperature, measured in Kelvin and *K* is the binding constant.

Circular Dichroism

The circular dichroism studies of lysozyme in presence of GATPT were carried out on JASCO J-815 spectropolarimeter. All the CD spectra were collected in a quartz cuvette of 1 cm path-length. The scan speed was 100 nm/min and response time of 1s for all measurements. Each spectrum was the average of 2 scans. All observed CD spectra were baseline subtracted for buffer and the CD results were expressed in terms of mean residue ellipticity (MRE) in deg cm^2 d mol^{-1}, which is given by the formula: [4]

$$MRE = \frac{\theta obs(mdeg)}{10 \text{ x } n \text{ x } C \text{ x } l} \qquad (10)$$

where θobs is the CD in milli-degree, n is the number of amino acid residues, l is the path length of the cell, and C is the molar concentration of protein. All spectra were smoothed by the Savitzky–Golay method with 10 convolution width. Alpha helical content was calculated from the MRE values at 222 nm using the following equation as described by Chen et al. [5]

$$\%\alpha - helix = \left(\frac{\text{MRE222} - 2{,}340}{30{,}300}\right) \text{x } 10 \qquad (11)$$

where MRE_{222} is the observed MRE value at 222 nm, 33000 is the MRE value of a pure α-helix at 222 nm.

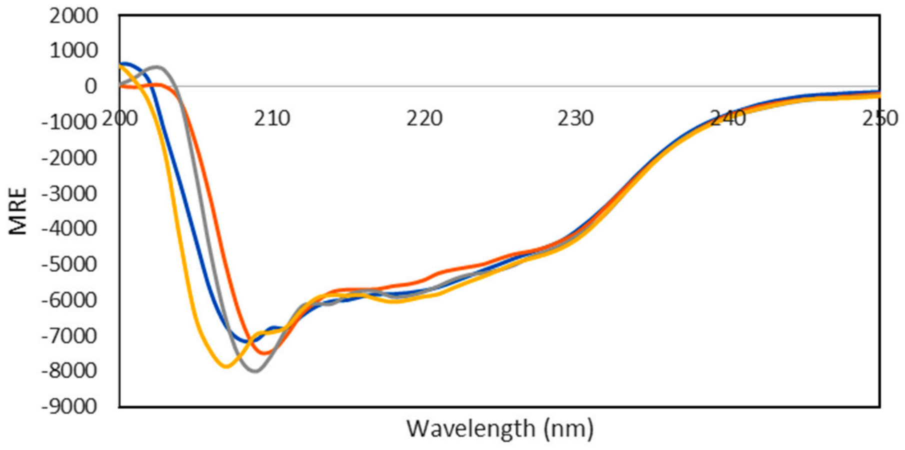

Fig. S1 Mean residual ellipticity (MRE) spectra of lysozyme in the absence and presence of different concentrations of GATPT at 25 °C, (Lysozyme) = 10^{-5} M (orange color), GATPT (grey, blue, red color: 5, 10, 20 µM respectively).

Molecular docking studies

The molecular docking studies were performed by using HEX 8.0 software [6] which is an interactive molecular graphics program used for the calculation and display of feasible docking modes of enzymes, DNA/RNA molecule. Hex 8.0. protein docking uses spherical polar Fourier Correlations and 3D FFT mode. It is the first protein docking program to be able to use modern graphics processor units (GPUs) to accelerate the calculations.

The structure of the complex was converted into PDB format from mol format by OPENBABEL (http://www.vcclab.org/lab/babel/). The structure of GATPT was optimized by applying the energy minimization through MM2 force field in Chemdraw 3D software. All rotatable bonds within the ligand were allowed to rotate freely and receptor was considered rigid. The crystal structure of HEWL (PDB id: 2LYZ) was retrieved from the protein data bank (http://www.rcsb.org./pdb). Visualization of minimum energy favorable docked poses was performed using Discovery Studio molecular graphics program.

References

1. S. Tabassum, S. Mathur, F. Arjmand, K. Mishra, K. Banerjee, Design, synthesis, characterization and DNA-binding studies of a triphenyltin (IV) complex of N-glycoside (GATPT), a sugar based apoptosis inducer: in vitro and in vivo assessment of induction of apoptosis by GATPT, Metallomics. 4 (2012) 205–217.
2. S. C. Gill, P. H. von Hippel, Calculation of protein extinction coefficients from amino acid sequence data, Anal. Biochem., 182 (1989) 319-326.
3. S. Das, S. Santra, M.A. Rohman, M. Ray, M. Jana, A.S. Roy, An insight into the binding of 6-hydroxyflavone with hen egg white lysozyme: a combined approach of multi-spectroscopic and computational studies, J. Biomol. Struct. Dyn. 37 (2018) 4019–4034.

4. M. S. Ali, H. A. Al-Lohedan, M.Z.A. Rafiquee, A. M. Atta, A. O. Ezzat, Spectroscopic studies on the interaction between novel polyvinylthiol-functionalized silver nanoparticles with lysozyme, Spectrochim. Acta A Mol. Biomol. Spectrosc., 135 (2015) 147–152.
5. Y. H. Chen, J. T. Yang, H. Martinez, Determination of the secondary structures of proteins by circular dichroism and optical rotator dispersion, Biochemistry. 11 (1972), 4120–4131.
6. D. Mustard, D.W. Ritchie, Docking essential dynamics eigenstructures, Proteins Struct. Funct. Bioinforma. 60 (2005) 269–274.
